# Digital Gender Gap in the Second Half of Life Is Declining: Changes in Gendered Internet Use Between 2014 and 2021 in Germany

**DOI:** 10.1093/geronb/gbad079

**Published:** 2023-05-22

**Authors:** Mareike Bünning, Anna Schlomann, Nicole Memmer, Clemens Tesch-Römer, Hans-Werner Wahl

**Affiliations:** German Centre of Gerontology, Berlin, Germany; Network Aging Research, Heidelberg University, Heidelberg, Germany; Network Aging Research, Heidelberg University, Heidelberg, Germany; German Centre of Gerontology, Berlin, Germany; Network Aging Research, Heidelberg University, Heidelberg, Germany

**Keywords:** Digital divide, Gender studies, Social change, Survey data

## Abstract

**Objectives:**

The main purpose of the study has been to examine changes in Internet use among men and women in 3 age groups (midlife, early old age, and advanced old age) between 2014 and 2021. We tested 2 hypotheses: The complementary hypothesis posits that online activities reproduce gender differences in offline activities. The compensatory hypothesis posits that women are catching up over time in male-typed activities as Internet access approaches saturation for both genders.

**Methods:**

We used representative, longitudinal data from the German Ageing Survey collected in 2014, 2017, 2020, and 2021 (*n* = 21,505, age range 46–90 years). We ran logistic regressions on Internet access and Internet use for 4 different gender-typed activities: social contact (female-typed), shopping (gender-neutral), entertainment (male-typed), and banking (male-typed).

**Results:**

Between 2014 and 2021, women drew level with men in Internet access. Gender differences in all 4 forms of Internet use declined considerably between 2014 and 2021. Women overtook men in using the Internet for social contact. In older age groups, men held the lead regarding online banking. During the coronavirus disease 2019 (COVID-19) crisis, women caught up to men in Internet use, especially for entertainment.

**Discussion:**

Overall time trends support the complementary hypothesis. By contrast, the finding that women have been catching up in some male-typed online activities during the COVID-19 pandemic supports the compensatory hypothesis.

Digital participation has become the *conditio sine qua non* for key aspects of social life in recent decades. Digital technologies facilitate communication, social engagement, and support, as well as access to information and services (Barbosa Neves et al., 2018; Cotten, 2017; Quan-Haase et al., 2016). This is especially true for individuals who have limited functional health and mobility (Quan-Haase et al., 2017). Digital participation is related to positive outcomes such as decreased loneliness (Schlomann et al., 2020), decreased depression (Cotten et al., 2014), increased subjective well-being (Nimrod, 2020), increased autonomy (Seifert et al., 2017), and positive self-perceptions of aging (Köttl et al., 2021) in older adults. The importance of digital participation constantly increases as many institutions such as shops, banks, and public authorities switch from onsite to online services (Jokisch & Göbl, 2022).

Digital participation is unevenly distributed, however. Especially women, lower-educated people, and (very) old people find themselves at a disadvantage. Social inequality affects both access to the Internet and its use. Unequal access to the Internet has been labeled the “first-level digital divide”; differential use of the Internet “second-level digital divide” (Friemel, 2016; Friemel & Signer, 2010). Previous research on the “grey divide” at large (Millward, 2003; Quan-Haase et al., 2018) as well as the role of education (Hargittai & Dobransky, 2017; König et al., 2018) has confirmed that older, less educated adults are less likely to have Internet access than younger, more educated adults (Huxhold et al., 2020 for Germany; Perrin, 2021 for the United States). With increasing diffusion of Internet access, inequalities in Internet use become a more prominent focus in the literature. Yet research on gender differences in Internet access and use in later adulthood has remained scarce and is needed because gender equality in “opportunities to thrive” is an important political goal (e.g., European Commission, 2020). For example, addressing digital gender disparities in certain online activities, such as the lower usage of online banking by women compared to men, may contribute to reducing economic opportunity gaps.

We argue in this paper that gendered Internet use represents a so far underdiscussed area for the gender and aging debate. Given that digital gaps are particularly prevalent in older age groups, we ask whether specific roles traditionally linked with gender translate to the digital world for adults in the second half of life. This is what the complementary hypothesis would suggest: gendered online activities reproduce gender differences that exist offline (Dannefer, 2020; van Deursen et al., 2015). By contrast, the compensatory hypothesis predicts that those who are less engaged in an activity offline may be more likely to adopt that activity online as a means of compensating for their disadvantages in the offline world. According to this perspective, the dissemination of Internet access may lead to the reduction of traditional gender roles.

In particular, we seek to answer the following questions: How do men and women differ in their *specific online activities* and how have these dynamics changed between 2014 and 2021? As Internet access is a precondition for online activities, we also investigate how gender differences in Internet access in later life changed between 2014 and 2021. Using data from the German Ageing Survey (DEAS), we have the unique opportunity to follow Internet access and use from 2014 to 2021, which enables us to test the complementary versus compensatory hypothesis of gendered behavior in the digital world. The DEAS is also unique in that it contains waves before and after the onset of the coronavirus disease (COVID-19) pandemic, making it possible to test for COVID-19 effects on changes in gender dynamics.

## Empirical Research on the Digital Gender Gap in Later Life

Studies have repeatedly shown that men of all ages more often have access to the Internet and engage in a greater range of online activities than women (e.g., Helsper, 2010; Joiner et al., 2015; Seifert et al., 2020). When looking at different types of Internet use, a more differentiated picture emerges. For instance, a male-dominated cluster of “practical” Internet users who mainly used the Internet for comparing and buying products as well as for financial activities emerged in a latent class analysis by van Boekel et al. (2017) in a sample of the Dutch population aged 65 years and older. Similarly, a study conducted in Germany involving participants aged 43 years and older showed that men were more likely than women to engage in both *online shopping* and *online banking* (Huxhold & Otte, 2019). Similarly, a British study on individuals aged 14 years and older found that men were more likely than women to engage in online shopping (Helsper, 2010). In some contrast, a recent study from Finland—a country with high levels of digitalization among the population in general and specifically among older adults (Pirhonen et al., 2020)—found no differences in online shopping between men and women aged 55–74, but did find that men were more likely than women to use mobile applications to pay for purchases (Kuoppamäki et al., 2017).

Regarding *entertainment*, most previous empirical studies have shown that men across the adult age range used the Internet for entertainment purposes more often than women (e.g., Helsper, 2010; Huxhold & Otte, 2019; Joiner et al., 2015; Kuoppamäki et al., 2017). Note, however, that van Deursen et al. (2015) found that even though men in the Netherlands were more likely than women to pursue leisure activities online, women were more likely than men to participate in online gaming in 2010 and just as likely as men to participate in online gaming in 2013 in a sample aged 16 and older.

Regarding *communication with family and friends*, van Boekel et al. (2017) found in a Dutch sample that older women were overrepresented in the cluster of “social” Internet users, who mainly used the Internet for reading, viewing, and posting messages, photos, or short films on social media. By contrast, Helsper (2010) found that men aged 14 and older were more likely than women to use the Internet for personal online communication (i.e., instant messaging, e-mailing, chat rooms, and making phone calls using the Internet). Finally, Huxhold and Otte (2019) found no evidence of gender differences in maintaining contact with family and friends online in a sample that included individuals from midlife to old age.

Taken together, available empirical research is mixed. Findings suggest that a male digital advantage continues to persist in some types of online activities, most notably online banking, but the evidence requires replication and extension to scrutinize possible historical changes in gendered Internet use across different types of online activities. This is particularly important as several of the studies cited above use samples that range from youth/young adulthood to advanced old age and do not pay attention to age differences.

## Understanding Gender Differences in Later-Life Internet Use: Conceptual Considerations

To analyze gender differences in Internet use, we drew on prominent analytical concepts in the social sciences, in particular, role theory and gendered work socialization.

### Gendered Social Roles and Stereotypes

Men and women differ in the roles they take on in society. Hence, many activities are perceived as stereotypically male or stereotypically female (Joiner et al., 2015; Kennedy et al., 2003; Meier et al., 2020). These roles and stereotypes may either facilitate or hinder use of the Internet in general. Especially in the early years of digitalization, Internet use was closely connected to computer use, a stereotypically male activity, which made it less appealing and accessible to women (Joiner et al., 2015; Kennedy et al., 2003). Moreover, women still perform the lion’s share of domestic and care work, which may limit the time they have available to acquire digital skills and pursue online activities (Calasanti & Slevin, 2001). When women gain access to the Internet, however, they may use this technology differently than men. Women usually are ascribed the role of “kinkeeper” (Rosenthal, 1985), which entails responsibility for maintaining social contact with family and friends (Helsper, 2010; Kennedy et al., 2003). Indeed, time use data show that women aged 45 years and older are more likely than men to make phone calls to friends and relatives and spend more time talking on the phone (Statistisches Bundesamt, 2015). This may carry over into the online world, leading women to play a more active role in online communication than men. Activities like banking or entertainment, in contrast, are considered stereotypically male. Regarding banking, men participate more on the stock market than women and score higher than women in terms of financial literacy (Almenberg & Dreber, 2015; Bucher-Koenen & Lusardi, 2011). Regarding entertainment, time use data show that men aged 46 and older spend more time watching TV than women (Statistisches Bundesamt, 2015). One might therefore assume that men also perform these activities more often online than women (Joiner et al., 2015; Kennedy et al., 2003). Other activities, by contrast, are less clearly gender-typed. For instance, according to time use data, men and women aged 46 and older spend roughly equal amounts of time on shopping (offline; Statistisches Bundesamt, 2015). According to gender role theory, one would expect that gender differences in online behavior reproduce and reinforce gender differences in offline activities, supporting predictions of what has been called the *complementary hypothesis* (Yu et al., 2015).

### Gendered Socialization Experiences

The digital gender gap may also be the product of differential exposure to technology. Men today are still more likely than women to work full-time continuously and to have technical occupations, and this disparity was even greater in earlier birth cohorts. This may have given men greater access to computers and the Internet as well as to technical experts who advised them in how to use technology, a resource that women might have lacked due to the long periods of time they often spent outside the labor force (Wasserman & Richmond-Abbott, 2005). It is noteworthy that work experience with computers correlates with Internet use later in life (König et al., 2018; Korupp & Szydlik, 2005).

The Internet may indeed set the stage for new socialization experiences that overcome gender stereotypes. Gaining access to the Internet may provide the opportunity to perform not only familiar activities but also new activities in the online world. This might lead to a convergence in Internet use and reduce the digital gender gap, supporting predictions of what has been called the *compensatory hypothesis* (Yu et al., 2015).

## Changes in the Digital Gender Gap

Digitalization of societies is a highly dynamic process that has led to a fluctuating digital gender gap over recent decades. Persistent socioeconomic inequalities between men and women may have caused gender differences in Internet access (van Deursen & van Dijk, 2015). As men often have greater financial resources than women, they may be more likely to own a computer and/or smartphone, giving them better access to the Internet. However, with increasing affordability of mobile devices, this factor may have lost its relevance. Hence, one could expect that the digital gender gap in access to the Internet has been closing over the last years.

Drawing on predictions of the compensatory and complimentary hypotheses, we argue that the general trend of a narrowing digital gender gap might not hold true for subdomains of Internet use. As the *complementary hypothesis* predicts that online activities reproduce or even reinforce gender differences that exist offline (Dannefer, 2020; van Deursen et al., 2015), a male advantage may be expected to dissipate in female-typed and gender-neutral but not in male-typed Internet activities. In contrast, the *compensatory hypothesis* predicts that those who are less engaged in an activity offline may be more likely to adopt that activity online as a means of compensating for their disadvantages in the offline world. Hence, according to the compensatory hypothesis, the Internet provides an opportunity for “new” gender behaviors and thus reduces gender differences across all types of online activities or fosters gender atypical behavior.

A closing of the gender gap is also expected by the *diffusion of innovations model* (Rogers, 2003). According to this model, new technology is first used by an avant-garde that often consists of a small, homogenous group of early adopters. Gradually, what was once a new technology becomes affordable and accessible to society at large. In the case of the Internet, young, highly educated men made up most of the early adopters, while women, older, and less educated people started using the Internet somewhat later (Huxhold et al., 2020). Hence, the overall prediction of diffusion theory would be that we should see a gradual gender convergence occurring earlier in younger and later in older age groups. From a life course perspective, this social change includes both the diffusion of the Internet to new populations and the fact that cohorts with greater exposure to technology reach older ages.

The COVID-19 pandemic may have given the dynamic process of societal digitalization an additional push by increasing the need to use the Internet to organize daily life (so-called period effect within the life course framework). As a result, access to the Internet may have increased for men and women, especially in older age groups, speeding up the diffusion process. It is unclear, however, whether the pandemic reinforced complementary or compensatory behavior relating to Internet use (Seifert et al., 2021).

## Research Questions and Hypotheses

Our primary research questions in this study were as follows:

First, how do men and women differ in their *specific online activities* and how have these dynamics changed between 2014 and 2021? Second, how has the COVID-19 pandemic affected gendered Internet use? The *complementary hypothesis* expects a narrowing of the gender gap or emerging female advantage, particularly in those areas that are traditionally female-typed such as online communication with family or gender-neutral such as shopping, and a persistent gender gap in male-typed activities such as online entertainment and online banking. The *compensatory hypothesis*, in contrast, expects a persistent male advantage or widening gender gap in online communication due to a disproportionate uptake of these activities by men, and a narrowing gender gap in online banking and online entertainment. Given the constraints on the analog world imposed by the COVID-19 pandemic, men and women might have tried to move their usual offline activities to an online environment (in line with the complementary hypothesis) or they might have developed new online behavior (in line with the compensatory hypothesis).

Before answering these research questions, we first examine gender differences in Internet access, to show to what extent the gender gap in Internet access indeed has closed over time.

## Method

### Data

The DEAS (Vogel et al., 2021) is a representative cohort-sequential study that integrates cross-sectional samples with longitudinal samples. Baseline samples of adults aged 40 and older living in private households in Germany (collected in 1996, 2002, 2008, and 2014) were drawn from a random sample of municipalities and stratified by age, gender, and region. Participants of the baseline samples were then contacted again for interviews for all subsequent waves unless they withdrew panel consent or dropped out of the sample due to death, permanent illness, or moving abroad. For this study, we used an unbalanced sample of the four most recent waves of DEAS (2014, 2017, 2020 [collected in June/July], and 2021 [collected from November 2020 to March 2021]) because these waves include information on Internet use for a wide range of activities. The sample hence covers two prepandemic periods as well as two measurement occasions during the COVID-19 pandemic.

Data collection consists of a personal interview and a self-administered questionnaire that was filled out by about 85% of respondents (Vogel et al., 2021; in summer 2020, only a self-administered questionnaire was distributed). Information on Internet access and use was collected with the self-administered questionnaire. We restricted the sample to respondents aged 46–90, as this age range was covered at all four time points. After listwise deletion (3.8% of cases, see Supplementary Table 1 for a comparison of cases with full information and those dropped by listwise deletion), sample size was 21,505 for the analyses on Internet access and ranged from 16,537 to 16,561 for the analyses on Internet use by those who had access.

### Measures

The dependent variables were Internet access and Internet use for four different purposes. Internet access was measured by the following question: “Do you have access to the Internet?,” with possible answers “yes, at work,” “yes, at home,” and “no.” If respondents had access to the Internet at home or at work, they were coded as having access.

Specific Internet use was measured by asking respondents with Internet access “How often do you use the Internet for the following purposes?” Purposes were: “contact with friends and relatives (e.g., e-mail, Facebook, chat, video telephony like Skype),” “search for new social contacts (e.g., friends, partner, like-minded persons),” “search for information (e.g., news, advisors, Wikipedia),” “banking business (e.g., online banking),” “entertainment (e.g., listening to music, watching films, playing games, watching TV),” “shopping (e.g., amazon, eBay, online pharmacy, food delivery),” and “creating own contents (e.g., texts, photos, music,up-loading videos for blogs, websites, online selling).” Answer options were (1) “daily,” (2) “several times a week,” (3) “once a week,” (4) “one to three times a month,” (5) “less often,” and (6) “never.” In the following, we report our analysis of one female-typed activity (contact with friends and relatives), two male-typed activities (banking and entertainment), and one gender-neutral activity (shopping; see Author Note 1).

We created a dummy variable for each purpose to analyze regular use. Respondents were coded as regularly using the Internet for a specific purpose if they reported doing an activity “once a week” or more often. We focused on contrasting regular Internet use with infrequent/no use because regular use is a prerequisite for digital social participation. Nevertheless, additional analyses distinguishing any use from never using the Internet for a specific purpose (see Supplementary Figures 1 and 2) revealed similar patterns. Mean values of all dependent variables by year are displayed in Table 1.

**Table 1.** Mean Values and Valid Cases of Dependent and Independent Variables by Year

	2014		2017		2020		2021	
	Mean	*N*	Mean	*N*	Mean	*N*	Mean	*N*
Dependent variables								
Has access to the Internet^a^	0.74	7,546	0.81	5,403	0.88	4,297	0.89	4,215
Regularly uses the Internet for…								
Contact with friends/relatives^a^	0.60	5,195	0.70	4,131	0.76	3,611	0.83	3,597
Banking^a^	0.37	5,182	0.40	4,122	0.51	3,613	0.51	3,597
Entertainment^a^	0.33	5,188	0.42	4,117	0.61	3,609	0.65	3,596
Shopping^a^	0.13	5,192	0.16	4,125	0.25	3,612	0.28	3,600
Independent variables								
Women^a^	0.52	7,546	0.52	5,403	0.51	4,297	0.52	4,215
Age								
46–60^a^	0.48	7,546	0.48	5,403	0.45	4,297	0.45	4,215
61–75^a^	0.35	7,546	0.34	5,403	0.37	4,297	0.36	4,215
76–90^a^	0.17	7,546	0.19	5,403	0.18	4,297	0.19	4,215
ISCED level of education								
Low^a^	0.09	7,546	0.09	5,403	0.06	4,297	0.10	4,215
Medium^a^	0.54	7,546	0.53	5,403	0.49	4,297	0.51	4,215
High^a^	0.37	7,546	0.38	5,403	0.44	4,297	0.39	4,215
Lives alone^a^	0.22	7,546	0.23	5,403	0.21	4,297	0.23	4,215
Health^b^	3.50	7,546	3.53	5,403	3.61	4,297	3.58	4,215
Working^a^	0.45	7,546	0.47	5,403	0.48	4,297	0.48	4,215
Standard of living^b^	3.83	7,546	3.97	5,403	3.89	4,297	4.10	4,215

*Notes*: All values are weighted. ISCED = International Standard Classification of Education.

^a^Dummy variable.

^b^Five-point scale (higher values indicate more positive ratings).

The main explanatory variables were gender (male vs female; see Author Note 2), age (distinguishing midlife [46- to 60-year-olds], early old age [61- to 75-year-olds], and advanced old age [76- to 90-year-olds]), and year (the main analyses included a dummy variable for each year; to estimate the impact of the COVID-19 pandemic, we entered year linearly and added a dummy for pandemic, with 2020 and 2021 coded as 1; 2014 and 2017 as 0). In addition, the models controlled for education (high, medium, and low according to the International Standard Classification of Education [ISCED] classification), living alone (dummy), self-rated health (5-point scale ranging from very bad to very good), being employed (dummy), and rating of current standard of living (5-point scale ranging from very bad to very good). Descriptive statistics on all explanatory variables by year are displayed in Table 1.

### Statistical Analysis

We proceeded in two steps. In the first, we aimed to provide a rich description of how gender differences in Internet access and use across the age range have changed over time. We ran logistic regressions and regressed each dependent variable on gender, age group, and their interaction terms, as well as the control variables. We also allowed for interactions between all of these terms by and year of assessment (dummy variables). From these models, we calculated predicted margins by gender and average marginal effects of gender across the three age groups by year of assessment and tested whether gender differences were statistically significant.

In a second step, we attempted to estimate the impact of the COVID-19 pandemic. We tested whether change in Internet access and use during the pandemic was above or below the change that would have been expected in case of linear development over time. To do so, we entered year as a linear variable and additionally included a dummy variable for the pandemic. For each of the three age groups, we regressed the dependent variables on gender, year of assessment (linear term), the interaction between gender and year of assessment, a dummy variable for the pandemic (yes = 1 in 2020 and 2021; no = 0 in 2014 and 2017), the interaction between gender and the pandemic, as well as control variables. We then obtained average marginal effects for the pandemic dummy for each gender in the Years 2020 and 2021.

All analyses were performed using Stata 15 (StataCorp, 2017) and Stata’s svy command to adjust for sample stratification, as well as for clustering of multiple observations per respondent. Poststratified, cross-sectional weights were used to adjust for nonresponse and panel attrition. We present results graphically; regression tables for step 1 can be found in Supplementary Table 2, and regression tables for step 2 in Supplementary Tables 3–7 (see Author Note 3).

## Results

We first look at changes in gendered Internet access in order to rule out the impact of gender-specific Internet access on gendered Internet use. Then, we empirically test the complementary versus compensatory hypothesis in respect to gendered Internet use.

### Gender Differences in Internet Access Between 2014 and 2021

**Figure 1.**
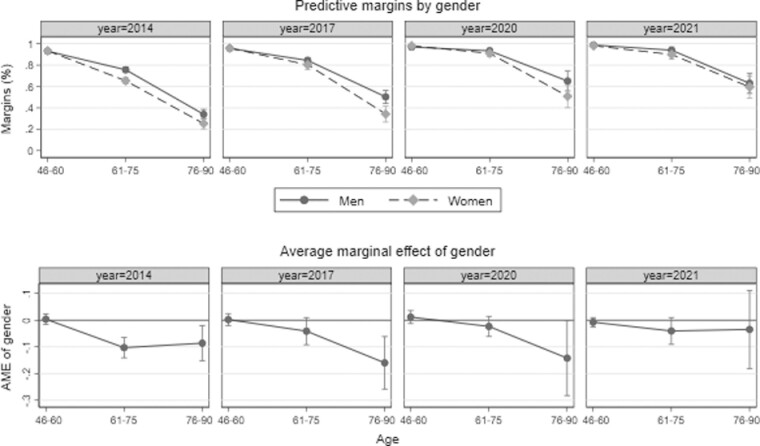
Internet access by gender and age between 2014 and 2021. Shown are predictive margins of Internet access by gender and age (upper panel) and average marginal effects of gender and age (lower panel). Estimates and 95% confidence intervals are displayed. Estimates are based on logistic regression analyses, controlling for education, household composition, self-rated health, subjective standard of living, and employment status. Interaction terms between all covariates and the years of data collection are included in the models (see Supplementary Table 3 for detailed information on regression coefficients). All analyses are adjusted for sample stratification and poststratified cross-sectional weights. The upper panel shows the proportion of men and women estimated to have Internet access at a certain age in a certain year of data collection. The lower panel shows whether Internet access differs significantly by gender. If confidence intervals are below (above) the 0-line, women were significantly less (more) likely than men to have access at a certain age in the respective year of data collection.

Figure 1 displays the results for Internet access. The upper panel shows predictive margins of Internet access by age group, year, and gender, that is, the predicted proportion of men and women who have access to the Internet. The lower panel shows the average marginal effect of gender. If the confidence interval of an effect is below 0, women at a certain age are significantly less likely to have access to the Internet than men. In 2014, there was a gender gap in Internet access in the population over 60 years of age, but no gender gap up to the age of 60. As indicated by predicted probabilities (upper panel of Figure 1), only 34% of men and 25% of women in advanced old age had access to the Internet in 2014. Over time, Internet access increased steadily among both older women and men, with the gender gap closing in 2021. As also indicated by predictive margins, almost 100% of individuals below 60 years of age had access to the Internet in 2021, compared to approximately 60% of those aged 76 years and older. This finding is in line with diffusion theory (Peres et al., 2010; Rogers, 2003), which postulates a narrowing gender gap across the full age range and not just among younger individuals over a long period of time.

### Gender Differences in Various Domains of Internet Use Between 2014 and 2021

#### Social contact with relatives and friends

**Figure 2.**
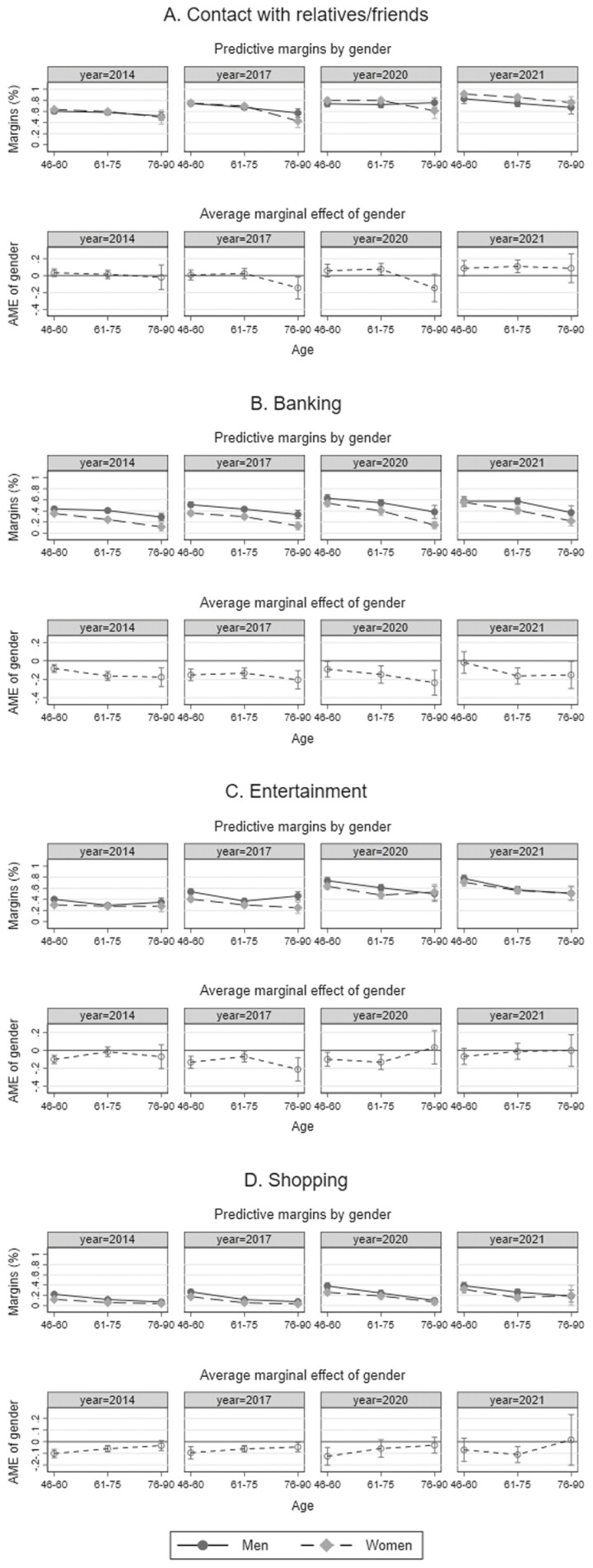
Regular Internet use by gender and age between 2014 and 2021 in the categories (A) contact with relatives and friends, (B) banking, (C) entertainment, and (D) shopping. Shown are predictive margins of Internet use by gender and age (first row in each panel) and average marginal effects of gender and age (second row in each panel). Estimates and 95% confidence intervals are displayed. Estimates are based on logistic regression analyses, controlling for education, household composition, self-rated health, subjective standard of living, and employment status. Interaction terms between all covariates and the years of data collection are included in the models (see Supplementary Table 3 for detailed information on regression coefficients). All analyses are adjusted for sample stratification and poststratified cross-sectional weights. The first row in each panel shows the proportion of men and women estimated to regularly use the Internet for the given purpose at a certain age in a certain year of data collection. The second row in each panel shows whether regular Internet use differs significantly by gender. If confidence intervals are below (above) the 0-line, women were significantly less (more) likely than men to have access at a certain age in the respective year of data collection.

Among those with Internet access, more than 60% of 46- to 60-year-old men and women regularly used the Internet for social contact with friends and relatives in 2014 compared to about 50% of 76- to 90-year-old men and women (upper panel of Figure 2A). Over time, regular Internet use for social contact increased in all age groups. By 2021, 91% of 46- to 60-year-old women and 83% of 46- to 60-year-old men regularly used the Internet to maintain contact with friends and relatives, as did 76% of 76- to 90-year-old women and 67% of 76- to 90-year-old men. As shown in the lower panel of Figure 2A, no statistically significant gender differences were found in 2014. Yet, in 2020 and 2021, regular Internet use for social contact was significantly more common among women than men among 61- to 75-year-olds, with similar trends (though not statistically significant at *p* < .05) in the other two age groups.

#### Online banking

As shown in Figure 2B, regular Internet use for banking was more common among men than women in all three age groups in the Years 2014–2020. In 2021, we still found a male advantage in older age groups but no gender gap among those younger than 60. Whereas 57% of 46- to 60-year-old men regularly engaged in online banking in 2021, the same was true of only 37% of 76- to 90-year-old men and 22% of 76- to 90-year-old women.

#### Online entertainment

As shown in Figure 2C, online entertainment was also more common among men than women in midlife up to 2020, but in 2021, gender differences were no longer statistically significant. In 2014, no gender difference was observed in the older age groups, but in 2017, men took the lead as more older men became regular users of online entertainment. By 2021, older women had also increased their online entertainment activities and the gender difference disappeared again in the older age groups. Whereas only around 30% of 76- to 90-year-old men and women used the Internet for entertainment regularly in 2014, figures increased to 51% by 2021.

#### Online shopping

As shown in Figure 2D, online shopping was significantly more common among men in midlife and early old age in 2014 and more common among men of all age groups in 2017. According to predicted probabilities, 22% of 46- to 60-year-old men and 12% of 46- to 60-year-old women regularly used the Internet for shopping in 2014, whereas the same was true for only 7% of 76- to 90-year-old men and 4% of 76- to 90-year-old women. Over time, Internet use for shopping increased among men and women of all age groups and in 2021, the gender difference was only statistically significant among 61- to 75-year olds. According to predicted probabilities, 39% of 46- to 60-year-old men and 32% of 46- to 60-year-old women regularly used the Internet for shopping in 2021, as did 18% of 90-year-old men and 20% of 90-year-old women.

### Effects of the COVID-19 Pandemic

#### Internet access during the pandemic

**Figure 3.**
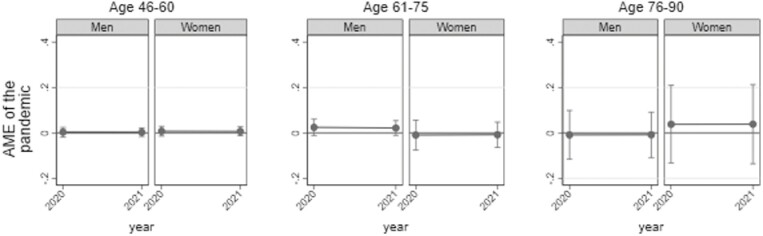
Effect of the pandemic on changes in Internet access by gender and age. Shown are average marginal effects of the coronavirus disease pandemic. Estimates and 95% confidence intervals are displayed. Estimates are based on logistic regression analyses, controlling for education, household composition, self-rated health, employment status, and subjective standard of living. Interaction coefficients between gender and year (linear) as well as gender and pandemic (dummy) are included in the models (see Supplementary Table 4 for detailed information on regression coefficients). The analyses are adjusted for sample stratification and poststratified cross-sectional weights. If confidence intervals are below (above) the 0-line, persons of the respective gender and age group were significantly less (more) likely to have access to the Internet during the pandemic than would have been expected given a linear time trend.

Figure 3 shows average marginal effects of the COVID-19 pandemic on Internet access by men and women. If the confidence interval of an effect was above 0, increases in Internet access during the pandemic would be significantly higher than expected by a linear time trend. Yet, given that the confidence intervals include 0 for men and women of all age groups, the COVID-19 pandemic did not accelerate or decelerate the rate of Internet access for either gender compared to what would have been expected as a linear time trend.

#### Internet use during the pandemic

**Figure 4.**
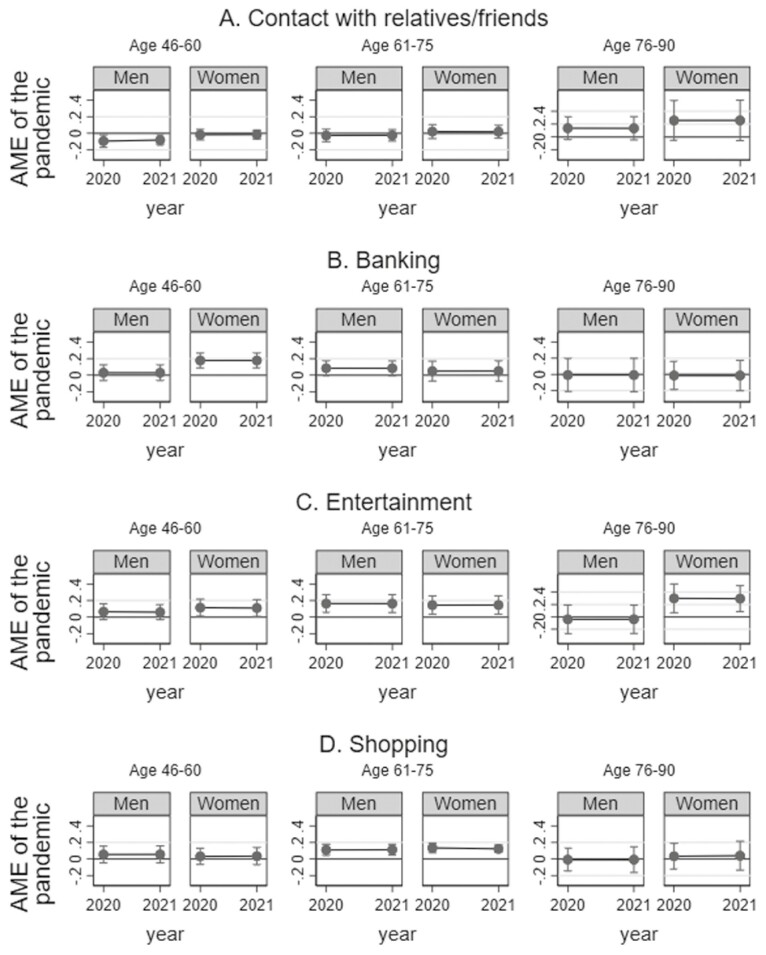
Effect of the pandemic on changes in regular Internet use by gender and age in the categories (A) contact with relatives and friends, (B) banking, (C) entertainment, and (D) shopping. Shown are average marginal effects of the coronavirus disease pandemic on regular Internet use. Estimates and 95% confidence intervals are displayed. Estimates are based on logistic regression analyses, controlling for education, household composition, self-rated health, subjective standard of living, and employment status. Interaction coefficients between gender and year (linear) as well as gender and pandemic (dummy) are included in the models (see Supplementary Tables 3–6 for detailed information on regression coefficients). All analyses are adjusted for sample stratification and poststratified cross-sectional weights. If confidence intervals are below (above) the 0-line, persons of the respective gender and age group were significantly less (more) likely to regularly use the Internet for the given purpose during the pandemic than would have been expected given a linear time trend.

During the pandemic, the predicted proportion of men aged 46–60 who reported regularly using the Internet for *contact with friends and relatives* was below what would have been expected in case of a linear time trend, whereas we found no statistically significant impact of the pandemic on Internet use for contact with relatives and friends and among women or older men (Figure 4A). Women between 46 and 60 years of age used the Internet for *banking* to a greater extent during the pandemic than what would have been forecasted by a linear time trend, but we found no evidence that the pandemic affected Internet use for banking among older women or men (Figure 4B). Women of all age groups used the Internet for *entertainment* to a greater extent during the pandemic, according to a test of deviation from a linear trend prediction (Figure 4C). Men between the ages of 61 and 75 also displayed elevated levels of regular Internet use for entertainment during the pandemic, whereas this was not the case for men in the two remaining age groups. Moreover, women and men between the ages of 61 and 75 were more likely to regularly use the Internet for *shopping* during the pandemic (Figure 4D).

## Discussion

This paper extends previous findings on the gendered digital divide in the second half of life (Huxhold et al., 2020; König et al., 2018) by presenting evidence that Internet access and use for all activities have increased among older men and women. In 2021, there was no longer a gender gap in access to the Internet from midlife to advanced old age.

More importantly, we empirically examined whether there are persistent gender differences in Internet use. This allowed us to provide not only a more qualified account of the gender divide in digital aging but also a better understanding of what impact the Internet has in enabling social participation. To conceptualize this impact, we argued that gender would moderate developments in Internet use as proposed by the complementary and the compensatory hypotheses (Peres et al., 2010; Yu et al., 2015).

Overall time trends were in line with the complementary hypothesis. With increasing diffusion of the Internet, gender differences narrowed and conformed more with gender-typed offline behavior. In 2014, men were still in the lead in Internet use, in that older men used the Internet more frequently than older women for banking (male-typed activity), and also for shopping (gender-neutral activity in the offline world), which replicated previous research (Helsper, 2010; Huxhold & Otte, 2019; Joiner et al., 2015), while we found no gender differences in social contact with friends and relatives and entertainment. By 2021, men led only in Internet use for banking, and only in the population over 60 years of age. There was no gender difference in Internet use for entertainment and shopping across the age range, and women had taken the lead in regular Internet use for contact with friends and relatives. Hence, while a female advantage emerged in the traditionally female-typed area of contact with friends and relatives, a gender gap in favor of men remained in the area of online banking, which is traditionally male-typed. By contrast, we found no gender differences in online shopping (gender-neutral offline) and online entertainment (male-typed offline).

During the pandemic, by contrast, we found behavior to be more in line with the compensatory hypothesis, as women in midlife disproportionately increased their engagement in male-typed online activities (banking and entertainment). Among those in early old age, both women and men engaged more in online entertainment and shopping during the pandemic. Women in advanced old age were also more likely to use the Internet for entertainment, but there was no evidence that men in the same age group changed their patterns of Internet use during the pandemic over and above what would have been expected given a linear time trend. Moreover, men in midlife were less likely to use the Internet for social contact than would have been expected otherwise. Hence, it seems that the pandemic contributed to narrowing gender gaps.

Tying our findings back into the debate on gendered aging in the digital world, we conclude that gender gaps continue to persist in some domains in older age groups, hinting at generational differences that intersect with gender. In addition, we found some evidence that gender stereotypes may even be reproduced by Internet use in later life. The only type of Internet use in which women surpassed men was in maintaining contact with family and friends, an activity that is clearly female-typed in the offline world as well. Hence, whereas the gender divide in Internet access has disappeared over time, gender differences in Internet use may be more persistent and may in fact strengthen gender stereotypes and support their role in shaping the ways people use the Internet (see also Joiner et al., 2015). At the same time, the pandemic seems to have served as a catalyst for women to catch up in some domains of Internet use, most notably entertainment, and thus to have reduced gender gaps in some male-typed areas.

### Implications

The results of our analyses show a general decrease of gender differences in Internet access and Internet use in the last years. These findings could inform the discourse on interventions for Internet access of older people. Identifying ways to help older adults successfully use and maintain use of digital tools in different areas of life may foster autonomy and inclusion in old age. As the digital gender gap is closing, societal gender stereotypes might be reinforced by focusing digital interventions especially (or only) on women. Though our findings do confirm that specifically targeting older women may be a fruitful approach in some domains of Internet use, such as online banking, our findings on gendered Internet use might inform interventions that address both women and men. Offering learning opportunities in a broad diversity of gendered Internet activities might influence, and possibly, change gendered behavior both on- and offline.

We framed this paper in the context of the multiple benefits associated with digital participation that have been corroborated by previous research (Barbosa Neves et al., 2018; Cotten, 2017; Cotten et al., 2014; Köttl et al., 2021; Nimrod, 2020; Quan-Haase et al., 2016; Schlomann et al., 2020; Seifert et al., 2017). It should be noted, however, that Internet use does not always yield positive outcomes and also bears risks such as Internet addiction (Frost & Rickwood, 2017; Kovačić Petrović et al., 2022). Even though problematic use is rare among older adults (Busch et al., 2021), previous studies indicate that Internet use for entertainment is related with increased loneliness, whereas other types of Internet use are related with decreased loneliness (Wang et al., 2018). Given gender differences in Internet use, future research should hence examine whether different types of Internet use are associated with different outcomes for men and women. Moreover, qualitative studies may provide additional insights into why older men and women continue to differ in their online behavior, what particular barriers they face, and what interventions may help to overcome them.

### Limitations

We were not able to analyze gender differences in digital skills or prior experience with the Internet, which would be an additional important dimension of the second-level digital divide between men and women. Testing the mechanisms that underlie gender differences was beyond the scope of this paper and should be addressed in future research. Moreover, the social change we observed likely results from both diffusion of Internet use to new populations and cohorts with greater exposure to technology reaching older ages. Disentangling these two processes may provide additional insights on how to support Internet use in older populations. Our study was based on a secondary analysis of data and was thus limited to the questions included in the DEAS. For example, specific information on different Internet activities has only been included in the DEAS since 2014. Further, the survey was conducted in Germany only. The dynamics of gender differences might differ in other countries, for example, in terms of the diffusion of Internet technology and the relevance of specific Internet activities for older men and women.

## Conclusion

This longitudinal study, covering four cross-sections across 8 years, found that the gender gap in Internet access and use narrowed over time. Furthermore, we found some evidence in favor of the complementary hypothesis: At the most recent measurement, there was still a higher proportion of men in older age groups using the Internet for some activities, but women had surpassed men in using the Internet for one female-typed activity: social contact with friends and relatives. This stresses the importance of focusing not only on Internet access but on specific online activities when addressing digital gender gaps, both in scientific research and in real-life interventions. Furthermore, the pandemic appears to have served as a catalyst for woman to catch up in some—especially male-typed—domains of Internet use. As such, the pandemic tended to decrease gender differences rather than increasing them. These patterns are more in line with the compensatory hypothesis. Still, additional data will be needed to further test the complementary and compensatory hypotheses in future research.

## Supplementary Material

gbad079_suppl_Supplementary_Appendix

## Data Availability

This publication is based on data from the German Ageing Survey (DEAS). Data and materials are available to the scientific community free of charge from the Research Data Center of the German Centre of Gerontology (data: dx.doi.org/10.5156/DEAS.1996-2021.M.001, materials: dx.doi.org/10.5156/DEAS.1996-2021.D.001). For reasons of data protection, a data distribution contract must be signed by data users before data can be provided. Analytic code needed to reproduce the analyses presented in this paper is deposited at https://osf.io/g37zf/?view_only=ae75d6c4e4074090a5cd5aa5fbb50c46.

## Author Notes

1. We do not present results on information search, search for new contacts, or creation of content. Empirically, the search for information is the most common type of Internet use, with little variation across sociodemographic groups (Huxhold & Otte, 2019; van Deursen et al., 2015), though men and women differ in the kinds of topics they seek information on (Helsper, 2010). Search for new contacts and creation of content are the least common activities of the seven items in the DEAS.

2. Since 2020, respondents have also been offered the answer category “divers” or nonbinary, but none of the respondents chose it.

3. Analytic code is available at https://osf.io/g37zf/?view_only=ae75d6c4e4074090a5cd5aa5fbb50c46.
