# Supplementary material for: Digital Gender Gap in the Second Half of Life Is Declining: Changes in Gendered Internet Use Between 2014 and 2021 in Germany
Source: J Gerontol B Psychol Sci Soc Sci. 2023 May 22;78(8):1386–95. doi: 10.1093/geronb/gbad079 (PMC10394992; doi:10.1093/geronb/gbad079)
Supplement: gbad079_suppl_Supplementary_Appendix [file gbad079_suppl_supplementary_appendix.docx]

**Online Appendix**

Supplement Table 1: Comparison of sample means among respondents with full information and respondents with item nonresponse

|  | Full information  sample | | Missing data  sample | |  |
| --- | --- | --- | --- | --- | --- |
|  |  |  |  |  |  |
|  | Mean | N | Mean | N |  |
| **Dependent variables** |  |  |  |  |  |
| Has access to the Internet | 0.82 | 21461 | 0.61 | 422 | * |
| Regularly uses the Internet for ... |  |  |  |  |  |
| ... contact with friends and relatives | 0.71 | 16534 | 0.68 | 344 |  |
| ... banking | 0.44 | 16514 | 0.37 | 335 | * |
| ... entertainment | 0.49 | 16510 | 0.57 | 335 | * |
| ... shopping | 0.20 | 16529 | 0.24 | 340 |  |
| **Independent Variables** |  |  |  |  |  |
| Women | 0.52 | 21461 | 0.62 | 845 | * |
| Age | 63.24 | 21461 | 70.31 | 845 | * |
| ISCED level of education |  |  |  |  |  |
| Low | 0.09 | 21461 | 0.15 | 841 | * |
| Medium | 0.52 | 21461 | 0.55 | 841 |  |
| High | 0.39 | 21461 | 0.31 | 841 | * |
| Lives alone | 0.22 | 21461 | 0.33 | 605 | * |
| Health | 3.55 | 21461 | 3.37 | 768 | * |
| Working | 0.47 | 21461 | 0.24 | 737 | * |
| Standard of living | 3.93 | 21461 | 3.72 | 780 | * |
| *N* | 21461 |  | 845 |  |  |

All values are weighted. *Difference statistically different at p<.05.

3.8 % of cases are lost due to listwise deletion. Item nonresponse is not completely missing at random. The cases that remain in the sample are younger and healthier than those with case nonresponse. They are more likely to be men, highly educated, not living alone, and working, and report a higher standard of living. They are also more likely to have access to the Internet and more likely to use the Internet for banking and entertainment, whereas they do not differ from cases with item nonresponse in the likelihood to regularly use the Internet for contact with friends and family or for online shopping. Nevertheless, biases and loss of power are likely to be inconsequential because the loss of cases is small and the relevant variables are adjusted for in the models (see Graham, John W. 2009. “Missing Data Analysis: Making It Work in the Real World.” Annual Review of Psychology 60:549–76. <https://doi.org/10.1146/annurev.psych.58.110405.085530>)

Supplementary Table 2: Regression of Internet access and regular Internet use for contacts with relatives/friends, banking, entertainment and shopping on gender, age, and year (log-odds)

|  | Access | Contacts | Banking | Entertainment | Shopping |
| --- | --- | --- | --- | --- | --- |
|  |  |  |  |  |  |
| Women | 0.06 | 0.14 | -0.36^***^ | -0.45^***^ | -0.75^***^ |
| Age | ref. | ref. | ref. | ref. | ref. |
| 61-75 | -0.88^***^ | -0.18 | -0.04 | -0.64^***^ | -0.69^***^ |
| 76-90 | -2.51^***^ | -0.51^**^ | -0.54^*^ | -0.44^*^ | -1.24^***^ |
| Gender*Age | ref. | ref. | ref. | ref. | ref. |
| Women*Age 61-75 | -0.63^**^ | -0.08 | -0.42^**^ | 0.37^*^ | -0.05 |
| Women*Age 76-90 | -0.54^*^ | -0.23 | -0.81 | 0.12 | 0.05 |
| Year | ref. | ref. | ref. | ref. | ref. |
| 2017 | -0.13 | 1.29^*^ | 0.27 | 0.68 | -2.12^*^ |
| 2020 | 0.41 | 1.22^*^ | 1.10 | -0.18 | 0.15 |
| 2021 | 1.56^*^ | 1.27^*^ | -0.67 | 1.88^**^ | -1.15 |
| Gender*Year | ref. | ref. | ref. | ref. | ref. |
| Women*2017 | -0.00 | -0.10 | -0.29^*^ | -0.09 | 0.17 |
| Women*2020 | 0.46 | 0.19 | -0.03 | -0.02 | 0.15 |
| Women*2021 | -0.58 | 0.66 | 0.28 | 0.09 | 0.42 |
| Age*Year | ref. | ref. | ref. | ref. | ref. |
| Age 61-75*2017 | 0.17 | -0.12 | -0.17 | 0.03 | -0.06 |
| Age 61-75*2020 | 0.85 | 0.11 | -0.44 | 0.08 | 0.03 |
| Age 61-75*2021 | -0.18 | -0.43 | 0.08 | -0.15 | 0.37 |
| Age 76-90*2017 | 0.34 | -0.23 | -0.07 | 0.20 | 0.14 |
| Age 76-90*2020 | 0.75 | 0.63 | -0.68 | -0.60 | -0.56 |
| Age 76-90*2021 | -0.39 | -0.55 | -0.10 | -0.63 | 0.52 |
| Gender*Age*Year | ref. | ref. | ref. | ref. | ref. |
| Women*Age61-75*2017 | 0.26 | 0.16 | 0.46^*^ | -0.15 | -0.22 |
| Women*Age61-75*2020 | -0.24 | 0.18 | 0.18 | -0.44 | 0.30 |
| Women*Age76-90*2021 | 0.55 | -0.00 | -0.20 | -0.06 | -0.34 |
| Women*Age76-90*2017 | -0.28 | -0.41 | 0.22 | -0.54 | -0.47 |
| Women*Age76-90*2020 | -0.67 | -0.81 | -0.12 | 0.48 | 0.15 |
| Women*Age76-90*2021 | 0.88 | -0.13 | 0.11 | 0.23 | 0.38 |
| ISCED level of education | ref. | ref. | ref. | ref. | ref. |
| Medium | 0.59^***^ | -0.14 | 0.54^*^ | 0.05 | -0.51 |
| High | 1.71^***^ | 0.34 | 1.00^***^ | 0.07 | -0.20 |
| Medium*2017 | -0.09 | -0.28 | 0.00 | -0.58 | 3.14^***^ |
| Medium*2020 | 0.26 | -0.42 | -0.34 | 0.21 | 0.78 |
| Medium*2021 | -0.40 | -0.36 | 0.49 | -1.08^*^ | 0.04 |
| High*2017 | -0.14 | -0.26 | 0.04 | -0.45 | 3.30^***^ |
| High*2020 | 0.18 | -0.48 | -0.33 | 0.37 | 0.96 |
| High*2021 | -0.41 | -0.13 | 0.47 | -1.12^*^ | 0.13 |
| Lives alone | -0.46^***^ | 0.24^*^ | -0.12 | 0.24^*^ | -0.17 |
| Lives alone*2017 | 0.08 | -0.17 | 0.06 | 0.03 | -0.57^*^ |
| Lives alone*2020 | -0.07 | -0.37 | -0.10 | -0.03 | -0.05 |
| Lives alone*2021 | -0.39 | -0.33 | -0.18 | -0.38 | 0.04 |
| Self-rated physical health | 0.20^***^ | 0.20^***^ | 0.08 | -0.03 | 0.00 |
| Health*2017 | 0.04 | -0.01 | -0.04 | 0.05 | -0.03 |
| Health*2020 | -0.13 | -0.02 | 0.06 | -0.02 | -0.13 |
| Health*2021 | 0.07 | -0.09 | 0.18 | 0.14 | 0.08 |
| Working | 1.03^***^ | -0.08 | 0.15 | -0.22 | 0.15 |
| Working*2017 | 0.24 | 0.16 | 0.03 | 0.36^*^ | 0.25 |
| Working*2020 | 0.62 | 0.06 | -0.40 | 0.32 | -0.09 |
| Working*2021 | 0.06 | -0.09 | -0.04 | 0.38 | 0.25 |
| Standard of living | 0.29^***^ | 0.19^***^ | 0.17^**^ | -0.12^*^ | 0.16^*^ |
| Standard of living*2017 | 0.11 | -0.12 | 0.03 | -0.03 | -0.21 |
| Standard of living*2020 | 0.09 | -0.03 | 0.04 | 0.29^**^ | 0.09 |
| Standard of living*2021 | 0.10 | 0.14 | 0.04 | 0.01 | 0.31 |
| _cons | -0.60^*^ | -1.06^***^ | -2.03^***^ | 0.26 | -1.67^***^ |
| *N* | 21461 | 16636 | 16612 | 16608 | 16629 |

All values are weighted.

^*^ *p* < 0.05, ^**^ *p* < 0.01, ^***^ *p* < 0.001

Supplementary Table 3: Regression of Internet access on gender, year and the Covid-19 pandemic in three age groups (log-odds)

|  | Age 46-60 | Age 61-75 | Age 76-90 |
| --- | --- | --- | --- |
|  |  |  |  |
| Woman | 40.56 | -168.45 | 42.19 |
| Year (linear) | 0.16^*^ | 0.16^***^ | 0.20^***^ |
| Woman*Year | -0.02 | 0.08 | -0.02 |
| Covid-19 Pandemic | 0.14 | 0.35 | -0.05 |
| Woman*Covid-19 Pandemic | 0.12 | -0.45 | 0.22 |
| ISCED level of education | ref. | ref. | ref. |
| Medium | -0.03 | 0.64^***^ | 0.69 |
| High | 1.82^***^ | 1.69^***^ | 1.61^***^ |
| Lives alone | -1.21^***^ | -0.25^*^ | -0.49^**^ |
| Self-rated physical health | -0.07 | 0.25^***^ | 0.32^***^ |
| Working | 1.26^***^ | 1.16^***^ | 0.29 |
| Standard of living | 0.40^***^ | 0.29^***^ | 0.38^**^ |
| _cons | -327.03^*^ | -330.67^***^ | -411.87^***^ |
| *N* | 6063 | 10044 | 5354 |

All values are weighted.

^*^ *p* < 0.05, ^**^ *p* < 0.01, ^***^ *p* < 0.001

Supplementary Table 4: Regression of using the Internet for contact with friends/relatives on gender, year and the Covid-19 pandemic in three age groups (log-odds)

|  | Age 46-60 | Age 61-75 | Age 76-90 |
| --- | --- | --- | --- |
|  |  |  |  |
| Woman | 45.04 | -70.79 | 136.01 |
| Year (linear) | 0.23^***^ | 0.13^**^ | 0.04 |
| Woman*Year | -0.02 | 0.04 | -0.07 |
| Covid-19 Pandemic | -0.64^*^ | -0.14 | 0.63 |
| Woman*Covid-19 Pandemic | 0.51 | 0.27 | 0.48 |
| ISCED level of education | ref. | ref. | ref. |
| Medium | -0.45 | -0.08 | -0.83 |
| High | 0.09 | 0.34 | -0.27 |
| Lives alone | -0.00 | 0.19 | -0.06 |
| Self-rated physical health | 0.14^**^ | 0.22^***^ | 0.20 |
| Working | 0.02 | -0.06 | -2.41 |
| Standard of living | 0.19^**^ | 0.20^***^ | 0.10 |
| _cons | -466.51^***^ | -256.66^**^ | -73.57 |
| *N* | 5682 | 8134 | 2820 |

All values are weighted.

^*^ *p* < 0.05, ^**^ *p* < 0.01, ^***^ *p* < 0.001

Supplementary Table 5: Regression of using the Internet for banking on gender, year and the Covid-19 pandemic in three age groups (log-odds)

|  | Age 46-60 | Age 61-75 | Age 76-90 |
| --- | --- | --- | --- |
|  |  |  |  |
| Woman | 140.22 | -77.82 | -63.97 |
| Year (linear) | 0.08^*^ | 0.04 | 0.08 |
| Woman*Year | -0.07 | 0.04 | 0.03 |
| Covid-19 Pandemic | 0.13 | 0.34 | -0.03 |
| Woman*Covid-19 Pandemic | 0.64^*^ | -0.13 | -0.07 |
| ISCED level of education | ref. | ref. | ref. |
| Medium | 0.93^*^ | 0.13 | 0.47 |
| High | 1.49^***^ | 0.47^*^ | 0.85 |
| Lives alone | -0.21 | -0.15 | -0.05 |
| Self-rated physical health | 0.14^*^ | 0.11 | -0.02 |
| Working | -0.10 | 0.15 | 0.00 |
| Standard of living | 0.18^**^ | 0.20^***^ | 0.24 |
| _cons | -158.77^*^ | -80.74 | -154.17 |
| *N* | 5679 | 8131 | 2799 |

All values are weighted.

^*^ *p* < 0.05, ^**^ *p* < 0.01, ^***^ *p* < 0.001

Supplementary Table 6: Regression of using the Internet for entertainment on gender, year and the Covid-19 pandemic in three age groups (log-odds)

|  | Age 46-60 | Age 61-75 | Age 76-90 |
| --- | --- | --- | --- |
|  |  |  |  |
| Woman | 32.00 | 23.16 | 381.92 |
| Year (linear) | 0.19^***^ | 0.08^*^ | 0.12 |
| Woman*Year | -0.02 | -0.01 | -0.19 |
| Covid-19 Pandemic | 0.31 | 0.66^**^ | -0.16 |
| Woman*Covid-19 Pandemic | 0.17 | -0.07 | 1.55 |
| ISCED level of education | ref. | ref. | ref. |
| Medium | -0.43 | -0.01 | -0.56 |
| High | -0.20 | -0.12 | -0.61 |
| Lives alone | -0.03 | 0.20 | 0.75^**^ |
| Self-rated physical health | 0.01 | 0.00 | 0.01 |
| Working | -0.11 | 0.14 | 1.46 |
| Standard of living | -0.05 | -0.03 | -0.08 |
| _cons | -377.79^***^ | -170.79^*^ | -251.22 |
| *N* | 5680 | 8124 | 2804 |

All values are weighted.

^*^ *p* < 0.05, ^**^ *p* < 0.01, ^***^ *p* < 0.001

Supplementary Table 7: Regression of using the Internet for shopping on gender, year and the Covid-19 pandemic in three age groups (log-odds)

|  | Age 46-60 | Age 61-75 | Age 76-90 |
| --- | --- | --- | --- |
| fruse_6_du |  |  |  |
| Woman | -123.02 | 283.19 | -173.08 |
| Year (linear) | 0.08^*^ | 0.04 | 0.16 |
| Woman*Year | 0.06 | -0.14 | 0.09 |
| Covid-19 Pandemic | 0.25 | 0.72^**^ | -0.06 |
| Woman*Covid-19 Pandemic | -0.07 | 1.06^*^ | 0.45 |
| ISCED level of education | ref. | ref. | ref. |
| Medium | 0.24 | 0.06 | -1.54^*^ |
| High | 0.75 | 0.31 | -1.21 |
| Lives alone | -0.38^*^ | -0.28 | 0.20 |
| Self-rated physical health | -0.02 | -0.02 | 0.08 |
| Working | 0.06 | 0.35^*^ | -0.44 |
| Standard of living | 0.26^**^ | 0.12 | 0.11 |
| _cons | -167.70^*^ | -77.08 | -326.98 |
| *N* | 5680 | 8135 | 2814 |

All values are weighted.

^*^ *p* < 0.05, ^**^ *p* < 0.01, ^***^ *p* < 0.001

Supplement Figure 1: Any Internet use by gender and age between 2014 and 2021 in the categories (A) contact with relatives and friends, (B) banking, (C) entertainment, and (D) shopping


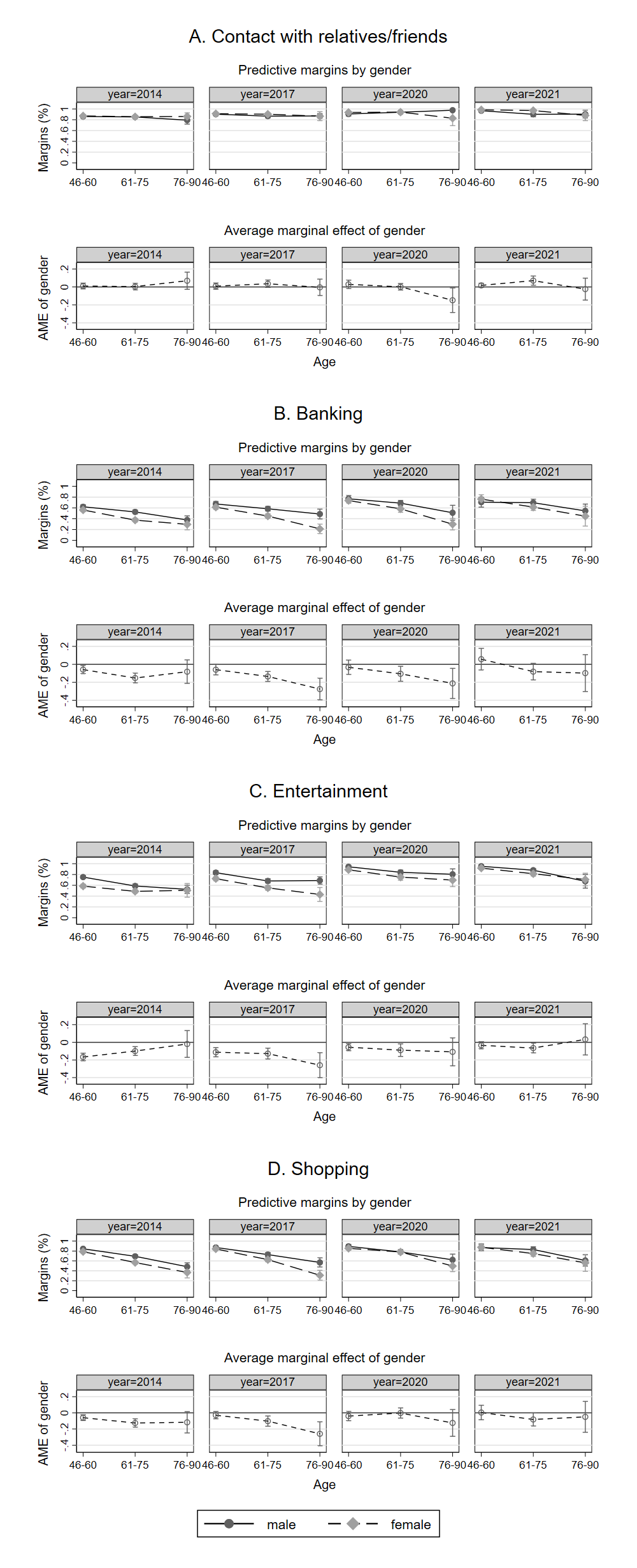


Note: Shown are predictive margins of Internet use by gender and age (first row in each panel) and average marginal effect (AME) of gender and age (second row in each panel) in the categories (a) contact with relatives and friends, (b) banking, (c) entertainment, and (d) shopping. Estimates and 95%-confidence intervals are displayed. Estimates are based on logistic regression analyses, controlling for education, household composition, self-rated health, employment status, and self-rated standard of living. Interaction terms between all covariates and the years of data collection are included in the models. All analyses are adjusted for sample stratification and post-stratified cross-sectional weights. The first row in each panel shows the proportion of men and women estimated to use the Internet at least sometimes for the given purpose at a certain age in a certain year of data collection. The second row in each panel shows whether Internet use differs significantly by gender. If confidence intervals are below [above] the 0-line, women were significantly less [more] likely than men to have access at a certain age in the respective year of data collection.

Supplement Figure 2: Effect of the pandemic on changes in Internet use by age and gender in the categories (a) contact with relatives and friends, (b) banking, (c) entertainment, and (d) shopping


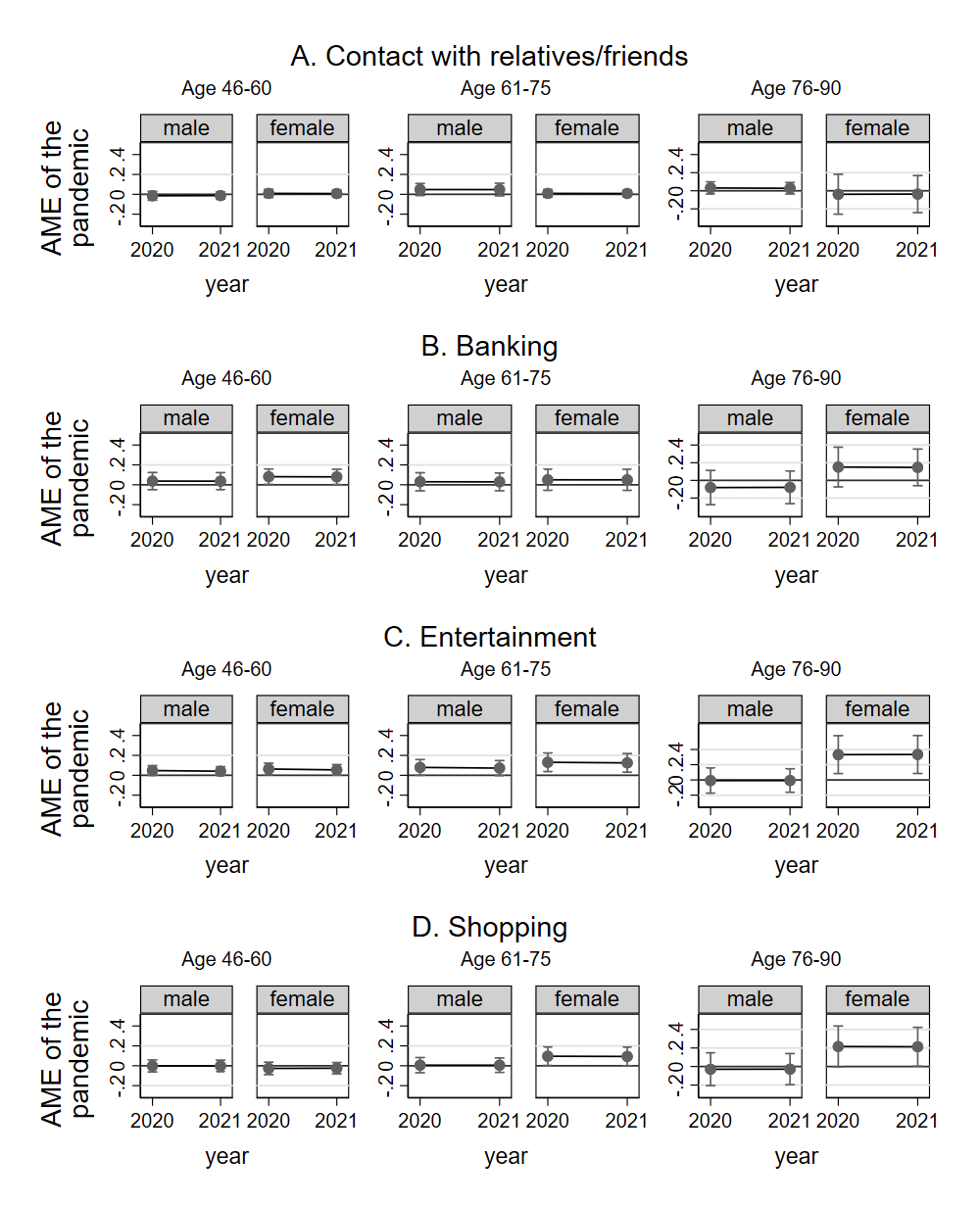


Note: Shown are average marginal effects of the COVID-19 pandemic on Internet use in the categories (a) contact with relatives and friends, (b) banking, (c) entertainment, and (d) shopping. Estimates and 95%-confidence intervals are displayed. Estimates are based on logistic regression analyses, controlling for education, household composition, self-rated health, employment status, and self-rated standard of living. Interaction coefficients between gender and year (linear) as well as gender and pandemic (dummy) are included in the models. All analyses are adjusted for sample stratification and post-stratified cross-sectional weights. If confidence intervals are below [above] the 0-line, persons of the respective gender and age group were significantly less [more] likely to use the Internet at least sometimes for the given purpose during the pandemic than would have been expected given a linear time trend.
